# Reversibility of Frail Phenotype in Patients with Inflammatory Bowel Diseases

**DOI:** 10.3390/jcm12072658

**Published:** 2023-04-03

**Authors:** Silvia Salvatori, Irene Marafini, Martina Franchin, Diletta Lavigna, Mattia Brigida, Chiara Venuto, Livia Biancone, Emma Calabrese, Diana Giannarelli, Giovanni Monteleone

**Affiliations:** 1 Gastroenterology Unit, Policlinico Universitario Tor Vergata, 00133 Rome, Italy; 2 Department of Systems Medicine, University of Rome Tor Vergata, Via Montpellier, 00133 Rome, Italy; 3 Facility of Epidemiology and Biostatistics, Fondazione Policlinico Universitario Agostino Gemelli IRCCS, 00168 Rome, Italy

**Keywords:** Crohn’s disease, ulcerative colitis, IBD, frailty, reversibility of frailty

## Abstract

It was recently reported that frailty status can negatively influence the clinical course of patients with inflammatory bowel diseases (IBDs). Our recent study demonstrated that 20% of patients with an IBD are frail, and disease activity increases the risk of frailty. In the present study, we prospectively monitored this subgroup of frail patients, assessed whether the frailty status was reversible, and analyzed factors associated with frailty reversibility. Of the sixty-four frail patients with IBD enrolled, five (8%) were lost during the follow-up period and one (2%) underwent a colectomy. Eleven out of the fifty-eight (19%) patients maintained a frail phenotype during a median follow-up of 8 months (range 6–19 months), and thirty-five (60%) and twelve (21%) became pre-frail or fit, respectively. A comparison of the 58 patients at baseline and at the end of the study showed that frail phenotype reversibility occurred more frequently in patients who achieved clinical remission. A multivariate analysis showed that the improvement of the frail phenotype was inversely correlated with the persistence of clinically active disease (OR:0.1; 95% CI: 0.02–0.8) and a history of extra-intestinal manifestations (OR:0.1; 95% CI: 0.01–0.6) and positively correlated with the use of biologics (OR: 21.7; 95% CI: 3.4–263). Data indicate that the frail phenotype is a reversible condition in most IBD patients, and such a change relies on the improvement in disease activity.

## 1. Introduction

There has recently been an enormous effort among clinicians to identify the factors influencing the natural course of chronic disorders. In this context, several studies have shown that patients with inflammatory bowel diseases (IBDs) have an enhanced risk of becoming frail, as these conditions are frequently associated with fatigue, sarcopenia, body weight loss, and serum hypoalbuminemia, which limit the patient’s autonomy and quality of life [1,2,3], facilitating the development of frailty status. Indeed, a higher frequency of frailty was seen in patients with IBD when compared to age- and sex-matched controls [1]. Frailty is defined as a reduction in or loss of physiologic reserve, and it increases the risk of complications in the long term [2]. Kochar and colleagues recently demonstrated that in IBD, frailty increases the risk of mortality, hospitalization, postoperative complications, and the risk of infections after therapy with immunosuppressors (ISSs) or biologic agents [1,3,4,5,6].

By using the Fried frailty phenotype [7], a score that has been widely validated [8,9,10] and is easy to use during scheduled outpatient visits, we recently showed [11] that nearly one-fifth of patients with IBD who were prospectively assessed in our tertiary referral center had a frail phenotype. Although frailty was more frequent in patients older than 60 years, univariate and multivariate analyses demonstrated that elderly age was not a risk factor for frailty. In contrast, our data indicated that patients with active IBD more frequently had a frail phenotype, and a multivariate analysis confirmed that active IBD, defined as the presence of clinical activity with biochemical (increased fecal calprotectin) and/or endoscopic activity, was an independent risk factor for the development of frailty. Overall, these findings suggest that in at least some subgroups of patients with IBD, frailty can be a consequence of the ongoing mucosal inflammation. If this is the case, then it is logical to hypothesize that resolution of the disease activity can ameliorate the frailty status. Support for such a hypothesis comes from a recent retrospective study which showed that frailty status could be improved in older patients treated with anti-TNF monoclonal antibodies [12]. However, considering the retrospective nature of the study, it remains unclear if those data are generalizable to the whole IBD population and whether other variables can influence the changes in frailty status. 

Here, we have monitored the dynamic changes in the frail phenotype in outpatients with IBD and analyzed predictive factors for such changes.

## 2. Materials and Methods

### 2.1. Study Population and Data Collection

This study is a follow-up study to our previous research, which aimed to assess the prevalence of the frail phenotype in outpatients with IBD and was conducted at the Tor Vergata Hospital in Rome (Italy). For the present study, we considered only those patients who had a frail phenotype at baseline according to the Fried frailty score [7]. Those patients were then followed up with at the indicated time points to ascertain any change in their frail status. The Fried frailty phenotype is a clinical score investigating five different domains: unintentional weight loss, self-reported exhaustion, slow walking speed, low physical activity, and weakness [7,11]. Patients with three or more of the five scoring criteria were defined as “frail”, while they were defined as “pre-frail” in presence of one or two of these criteria. Patients without any of these criteria were considered “fit”. Before enrolment, written informed consent was obtained from all the participants.

Information about age, gender, type of IBD, body mass index (BMI), history of extraintestinal manifestations (EIMs), history of steroid dependence (defined as patients with relapse within 3 months of stopping steroids) or steroid resistance (defined as patients with active disease despite the administration of prednisolone at up to 1 mg/kg/day for 4 weeks) was collected from clinical charts. A clinically active disease was defined as patients with a partial Mayo score [13] ≥2 in patients with ulcerative colitis (UC) and with a Harvey Bradshaw Index (HBI) [14] ≥5 in patients with Crohn’s disease (CD). Information about current therapy at baseline and during the follow-up period, the Charlson comorbidity index (CCI), and all reported comorbidities, diagnosed by referral specialists, were collected. We excluded from the study underage patients and patients unable to understand the informed consent (e.g., due to linguistic barriers). The study was approved by the local Ethics Committee (N° 3222).

### 2.2. Statistical Analysis

Qualitative data were expressed as numbers and proportions (%), and quantitative data were expressed as the median (range) after checking for a normal distribution of the data. The difference between the qualitative and quantitative variables at baseline and the end of the study was assessed using the McNemar test and Wilcoxon rank test, respectively. The patients’ characteristics were compared using Fisher’s exact test for the categorical variables and the Mann–Whitney U test for the continuous variables. Binomial logistic regression was performed, and parameters with *p* < 0.05 on a univariate analysis were used to perform a multivariate logistic regression analysis. The results of the logistic regression analysis were expressed using an odds ratio (OR) and 95% confidence intervals (CIs) with *p* values. Statistical analyses were performed using SPSS, version 26, and GraphPad Prism, version 9.0.

## 3. Results

### 3.1. Study Population and Changes in Frail Phenotype

**Figure 1:**
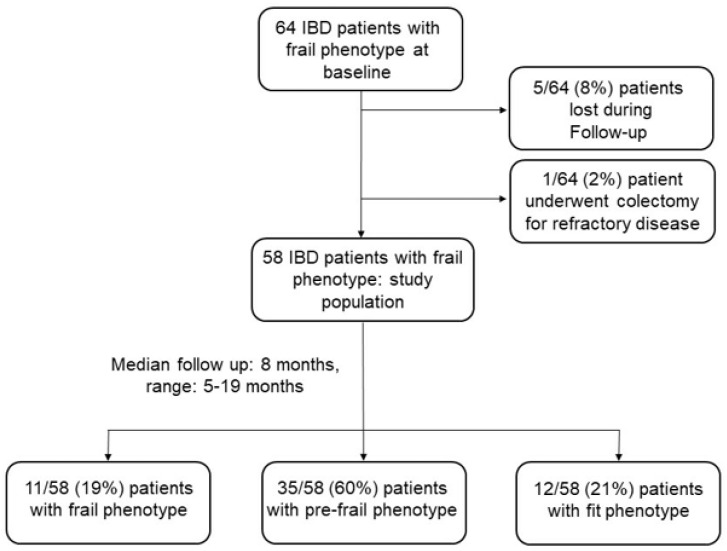
Flowchart of patients with a frail phenotype enrolled in the study. Data regarding the frequency of frail, pre-frail, or fit phenotypes at the end of the study are shown.

**Figure 2:**
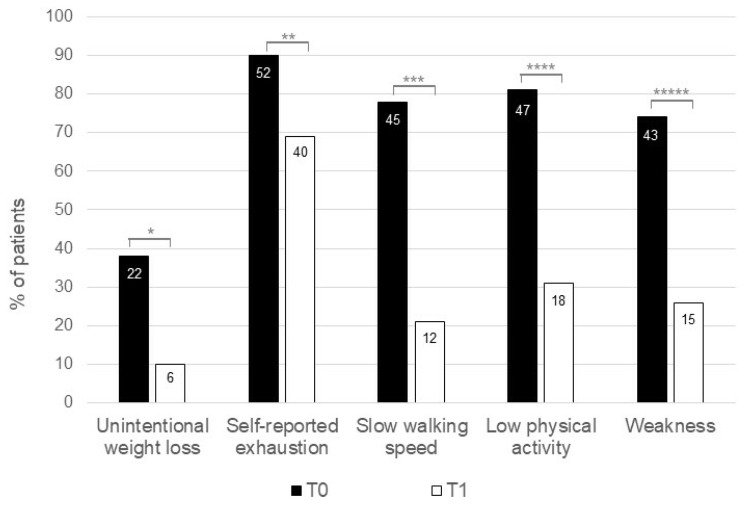
Changes in each component of the Fried frailty phenotype between the baseline and the end of the study. Difference assessed with the McNemar test for unintentional weight loss, self-reported exhaustion, slow walking speed, low physical activity, and weakness was statistically significant for each item: * *p* < 0.0001, ** *p* = 0.012, *** *p* < 0.0001, **** *p* < 0.0001, ***** *p* < 0.0001.

Sixty-four patients with IBD and a frail phenotype at baseline were followed up with for a median time of 8 months (range: 5–19 months). Five out of these sixty-four patients (8%) were lost during the follow-up period, and one patient underwent a colectomy for refractory disease. Therefore, our study population comprised 58 patients with IBD (31 with CD and 27 with UC; median age of 54 years, range: 18–78). The demographic and clinical characteristics of these patients are shown in Table 1. A comparison of the demographic and clinical characteristics of the 58 patients with IBD at baseline and at the end of the study demonstrated that the percentage of patients with the clinically active disease was significantly greater at baseline compared to at the end of the study (Table 1). During the follow-up period, 38 out of 58 patients (66%) received biological agents (Table 1). In particular, 21 out of 38 patients (55%) were receiving anti-TNF agents, 6 out of these at an optimized dose (10 mg/kg every 8 weeks) and the others at a standard dose (5 mg/kg every 8 weeks). Thirteen out of thirty-eight patients (34%) were on α4ϐ7 anti-integrin therapy (Vedolizumab), two patients at an optimized dose (300 mg every 4 weeks), and the others on a standard dose (300 mg every 4 weeks). Four out of thirty-eight patients (11%) were in therapy with anti-p40 IL12-23 (Ustekinumab) with a standard maintenance dose (90 mg every 8 weeks). At the end of the study, 11 out of 58 patients (19%) (six with CD and five with UC) maintained a frail phenotype, while 35 (60%) (20 with CD and 15 with UC) and 12 out of 58 patients (21%) (five with CD and seven with UC) became pre-frail and fit, respectively (Figure 1). An analysis of the frequencies of each component of the Fried frailty phenotype showed that, at baseline, the self-reported exhaustion, low physical activity, slow walking speed, and weakness were the most prevalent items. The frequency of such items, as well as of the changes in unintentional weight loss, was significantly reduced at the end of the study (Figure 2).

### 3.2. Reversibility of Frail Phenotype Occurs More Frequently following Induction of Clinical Remission

**Figure 3:**
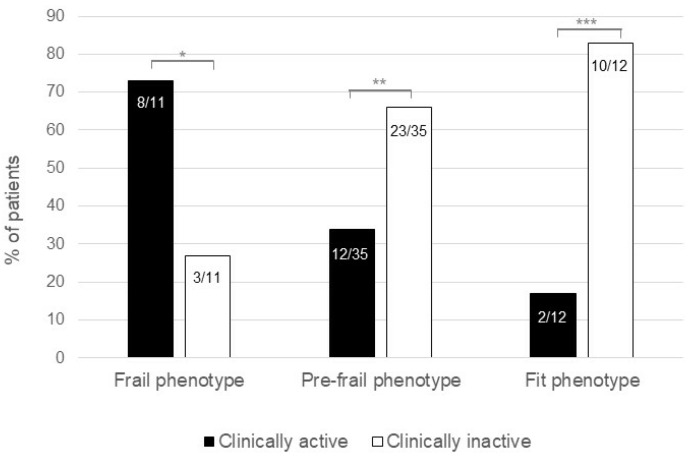
Correlation between changes in the frail phenotype at the end of the study and clinical disease activity in the study population. The percentage of patients with a frail phenotype and active IBD at the end of the study was significantly higher compared to patients with a frail phenotype and inactive IBD (8/11, 73% vs. 3/11, 27%; *p* < 0.0001), while pre-frail and fit patients more frequently had an inactive disease (*p* < 0.0001 in both cases). * *p* < 0.0001, ** *p* < 0.0001, *** *p* < 0.0001.

At the end of the study, the clinically active disease was more frequent in patients who maintained a frail phenotype (8/11, 73%) when compared to patients who became pre-frail or fit (12/35, 34% and 2/12, 17%, respectively) (Figure 3). A comparison of the clinical and demographic characteristics between patients who maintained a frail phenotype with those who became fit at the end of the study showed that the clinically active disease was more frequent in patients with a frail phenotype (8/11, 73%) than in fit patients (2/12, 17%; *p* = 0.012) (Table 2). In contrast, patients with a fit phenotype were more frequently in therapy with biologic agents (3/11, 27%) when compared to frail patients (9/12, 75%; *p* = 0.039) (Table 2).

Further analysis of the variables associated with frail status changes showed that patients who remained frail at the end of the study more frequently had a history of EIMs and were more clinically active when compared to those who exhibited an improvement in their frail status (Table 3). In contrast, the persistence of the frail phenotype was less frequent in patients who received therapy with steroids, ISS, or biologics (Table 3).

### 3.3. Predictive Factors for Phenotype Improvement

A univariate analysis with binomial logistic regression showed that a history of EIMs (OR 0.2; 95% CI: 0.04–0.7) and clinically active IBD (OR 0.2; 95% CI: 0.03–0.6) were risk factors for the persistence of the frail phenotype during the follow-up period, while current therapy with biologic agents was a predictive factor for an improvement in the frail phenotype (OR 7.8; 95% CI: 1.9–40.2). A multivariate analysis performed with multinomial logistic regression confirmed such associations (Table 4).

## 4. Discussion

This study is a follow-up of our recent prospective study, showing that in IBD, the frail phenotype is associated with disease activity. Here, we assessed whether the resolution of IBD clinical activity reverts the frail phenotype. Our data indicate that, after a median follow-up of 8 months, nearly 20% of the patients continued to be frail, while in the remaining patients there was an improvement in the frail status, and one-fifth of the latter became fit. The changes in the frail phenotype were paralleled by improvements in the clinical activity of the patients. Since patients with active IBD at baseline were treated with drugs that are known to control the ongoing mucosal inflammation (i.e., steroids or oral or rectal mesalamine in UC), the improvement in the frailty status is likely to be secondary to the attenuation/resolution of the gut inflammation. It is also noteworthy that no specific approach was made to correct the frail status of our patients (e.g., nutritional support, physiotherapy). Indeed, the resolution of clinical activity was associated with a transition from frail to fit status in most patients, while those who were clinically active at the end of the study remained prevalently frail.

A potential deleterious effect of disease activity on frailty was recently documented in patients affected by rheumatoid arthritis (RA). For example, in a cross-sectional analysis of Japanese RA patients, Tada and colleagues reported that the disease activity and modified health assessment questionnaire were higher in the frailty group, while only a small fraction of patients in remission were frail. However, following a multivariate analysis, matrix metalloproteinase 3 was the only independent factor for frailty [15]. In a monocentric cross-sectional study on 100 patients with RA aged 18–65 years, Haider and colleagues showed that compared to fit patients, the pre-frail/frail individuals demonstrated significantly higher disease activity, and the multivariable analysis showed that higher disease activity, unemployment, higher pain intensity, and long-lasting disease were correlated with a higher frailty score [16]. Finally, in another study of 375 patients with RA between 40 and 79 years of age, depression, physical function, and disease activity were associated with frailty [17].

The analysis of factors associated with frailty changes showed that at the end of our study, fit patients were more likely to be in treatment with biologics, while frail patients more frequently had a clinically active IBD and a history of EIMs. Finally, univariate and multivariate analyses showed that both histories of EIMs and clinically active IBD were independent risk factors for the persistence of frailty, while current treatment with biologics was a predictive factor for an improvement of the frail phenotype. Overall, these findings confirm and expand on recent data showing that the response to anti-TNF monoclonal antibodies is an important determinant in reducing post-treatment frailty in patients with CD or UC, with higher evidence found in older patients [12]. Indirect support for the positive impact of therapy on frailty also comes from several studies that demonstrated a correlation between frailty and circulating markers of inflammation in several conditions [18,19,20,21]. Moreover, in patients with RA, the effectiveness of biological disease-modifying antirheumatic drugs was associated with improvements in clinical variables (i.e., physical activity, nutritional status, and body weight) which influence the frail status [22].

We are aware that our study has some limitations. First, the study was conducted on a small group of patients because we enrolled and monitored for frailty changes only those who were identified as frail in our previous study. Second, we were able to document a significant reduction in the five components of the Fried phenotype during the follow-up period, but this score does not capture some relevant aspects of the overall frailty status (i.e., nutritional and psychological status). Therefore, our data deserve confirmation in future studies with validated scores that encompass all the components of frailty. Strictly related to this point is also the impossibility to quantify the frail status, making it difficult to understand whether the severity of frailty at baseline is a factor influencing the dynamic changes in response to IBD-related therapy. Third, the disease activity was defined using clinical scores, as biochemical (fecal calprotectin) and endoscopic data were absent in most patients. Therefore, it remains unclear whether the clinically active disease was always sustained by active intestinal inflammation. Finally, the short period of follow-up did not allow us to ascertain whether further relapses of IBD can re-induce frailty in patients who became fit during the study as well as whether some forms of frailty remain stable over time independently of the ongoing mucosal inflammation.

In conclusion, our study shows that frailty status can be improved or reverted in most patients with IBD, particularly after effective treatment. The demonstration that in a small subgroup of patients, the persistence of frailty is associated with a history of EIMs suggests further studies to ascertain the impact of various forms of frailty on specific IBD outcomes.

## Figures and Tables

**Table 1:** Demographic and clinical characteristics of the enrolled patients at baseline (T0) and at the end of the study (T1). CD: Crohn’s disease; UC: ulcerative colitis; BMI: body mass index; EIMs: extraintestinal manifestations; ISS: immunosuppressors.

Characteristics (N = 58)	T0	T1	*p*-Value
Age (years), median [range]	54 [18–78]	-	-
Female gender, n (%)	36 (62)	-	-
CD, n (%)	31 (53)	-	-
CD localization			
L1, n (%)	19/31 (61)	-	-
L2, n (%)	4/31 (13)	-	-
L3, n (%)	8/31 (26)	-	-
L4, n (%)	0	-	-
CD behaviour			
B1, n (%)	11/31 (35)	-	-
B2, n (%)	16/31 (52)	-	-
B3, n (%)	4/31 (13)	-	-
UC, n (%)	27 (47)	-	-
UC extension			
E1, n (%)	2/27 (7)	-	-
E2, n (%)	10/27 (37)	-	-
E3, n (%)	15/27 (56)	-	-
Age at diagnosis			
A1, n (%)	5 (9)	-	-
A2, n (%)	34 (58)	-	-
A3, n (%)	19 (33)	-	-
Perianal Disease, n (%)	11 (19)		
BMI (kg/m^2^), median [range]	25 [16–42]	24 [15–41]	0.09
History of EIMs, n (%)	19 (33)	-	-
History of steroid dependance/resistance, n (%)	26 (45)	-	-
Clinically active disease, n (%)	45 (78)	22 (38)	**<0.0001**
Current therapy with steroids, n (%)	10 (17)	4 (7)	0.109
Current therapy with biologic agents, n (%)	36 (62)	38 (66)	0.77
Current therapy with ISS, n (%)	2 (3)	2 (3)	>0.99
Current therapy with mesalamine, n (%)	37 (64)	38 (66)	>0.99
Charlson comorbidity index, median [range]	1 [0–5]	1 [0–5]	0.25
Psychiatric diseases, n (%)	10 (17)	12 (21)	0.69
Osteoarticular diseases, n (%)	13 (22)	15 (26)	0.69
Heart failure, n (%)	1 (2)	1 (2)	>0.99
Pneumological diseases, n (%)	5 (9)	5 (9)	>0.99
Neurodegenerative diseases, n (%)	1 (2)	1 (2)	>0.99
Post-COVID fatigue, n (%)	2 (3)	2 (3)	>0.99

**Table 2:** Demographic and clinical characteristics of patients with frail phenotype compared to patients with fit phenotype at the end of the study (T1). CD: Crohn’s disease; UC: ulcerative colitis; BMI: body mass index; EIMs: extraintestinal manifestations; ISS: immunosuppressors.

Characteristics at T1	Frail Phenotype (N = 11)	Fit Phenotype (N = 12)	*p*-Value
Age (years), median [range]	59 [27–78]	57.5 [24–71]	0.67
Female gender, n (%)	8 (73)	8 (67)	>0.99
CD, n (%)	6 (55)	5 (42)	0.68
UC, n (%)	5 (45)	7 (58)	0.67
Duration of disease (months), median [range]	180 [24–492]	168 [36–432]	0.88
BMI (kg/m^2^), median [range]	25 [18–31]	24 [16–33]	0.78
History of EIMs, n (%)	7 (64)	3 (25)	0.10
History of steroid dependence/resistance, n (%)	4 (36)	6 (50)	0.68
Clinically active disease, n (%)	8 (73)	2 (17)	**0.012**
Current therapy with steroids, n (%)	0 (0)	1 (8)	>0.99
Current therapy with biologic agents, n (%)	3 (27)	9 (75)	**0.039**
Current therapy with ISS, n (%)	0	0	-
Current therapy with mesalamine, n (%)	7 (64)	11 (92)	0.15
Charlson comorbidity index, median [range]	3 [0–5]	1.5 [0–4]	0.31
Psychiatric diseases, n (%)	2 (18)	1 (8)	0.59
Heart failure, n (%)	0	0	-
Pneumological diseases, n (%)	1 (9)	0	0.48
Neurodegenerative diseases, n (%)	0	1 (8)	>0.99
Post-COVID fatigue, n (%)	1 (9)	1 (8)	>0.99

**Table 3:** Demographic and clinical characteristics of patients with IBD who improved their phenotype at the end of the study (T1) compared to patients with IBD who maintained a frail phenotype at T1. CD: Crohn’s disease; UC: ulcerative colitis; BMI: body mass index; EIMs: extraintestinal manifestations; ISS: immunosuppressors.

Characteristics at T1	Improved Frail Phenotype (N = 47)	Persistence of Frail Phenotype (N = 11)	*p*-Value
Age (years), median [range]	54 [18–71]	59 [27–78]	0.27
Female gender, n (%)	28 (60)	8 (73)	0.07
CD, n (%)	25 (53)	6 (55)	0.88
UC, n (%)	22 (47)	5 (45)	0.88
Duration of disease (months), median [range]	156 [10–624]	180 [24–492]	0.99
BMI (kg/m^2^), median [range]	24 [15–41]	23.5 [20–33]	0.83
History of EIMs, n (%)	12 (26)	7 (64)	**0.03**
History of steroid dependence/resistance, n (%)	22 (47)	4 (36)	0.74
Clinically active disease, n (%)	14 (30)	8 (73)	**<0.0001**
Current therapy with steroids, n (%)	4 (9)	0	**0.001**
Current therapy with biologic agents, n (%)	35 (74)	3 (27)	**<0.0001**
Current therapy with ISS, n (%)	2 (4)	0	**0.048**
Current therapy with mesalamine, n (%)	31 (66)	7 (64)	0.88
Charlson Comorbidity Index, median [range]	1 [0–5]	3 [0–5]	0.08
Psychiatric diseases, n (%)	9 (19)	2 (18)	0.90
Heart failure, n (%)	1 (2)	0	0.50
Pneumological diseases, n (%)	5 (11)	1 (9)	0.81
Neurodegenerative diseases, n (%)	1 (2)	0	0.50
Post-COVID fatigue, n (%)	1 (2)	1 (9)	0.06

**Table 4:** Predictive factors for improvement of frail phenotype at the end of the study. OR: odds ratio; CI: confidence interval; CD: Crohn’s disease; UC: ulcerative colitis; BMI: body mass index; EIMs: extraintestinal manifestations; ISS: immunosuppressors.

	Univariate Analysis		Multivariate Analysis	
Risk Factors	OR (95% CI)	*p*-Value	OR (95% CI)	*p*-Value
Age (years)	0.97 (0.9–1.0)	0.26	-	-
Female gender	0.6 (0.1–2.2)	0.41	-	-
CD	0.95 (0.2 3–6)	0.94	-	-
UC	1.1 (0.3–4.1)	0.95	-	-
Duration of disease (months)	1.0 (0.9–1.0)	0.96	-	-
BMI (kg/m^2^)	1.0 (0.9–1.2)	0.93	-	-
History of EIMs	**0.2 (0.04–0.7)**	**0.02**	**0.1 (0.02–0.8)**	**0.04**
History of steroid dependence/resistance	1.5 (0.4–6.5)	0.52	-	-
Clinically active disease	**0.2 (0.03–0.6)**	**0.01**	**0.1 (0.01–0.6)**	**0.02**
Current therapy with steroids	1.2 (0.2–24.1)	0.88	-	-
Current therapy with biologic agents	**7.8 (1.9–40.2)**	**0.007**	**21.7 (3.4–263)**	**0.004**
Current therapy with ISS	0.7 (0.08–14.6)	0.75	-	-
Current therapy with mesalamine	1.1 (0.3–4.3)	0.88	-	-
Charlson comorbidity index	0.7 (0.4–1.1)	0.10	-	-
Psychiatric diseases	1.1 (0.2–7.8)	0.94	-	-
Heart failure	0.4 (0.04–10.1)	0.52	-	-
Pneumological diseases	1.2 (0.2–24.1)	0.88	-	-
Neurodegenerative diseases	0.4 (0.04–10.1)	0.52	-	-
Post-COVID fatigue	0.2 (0.02–1.8)	0.13	-	-

## Data Availability

The data presented in this study are available on request from the corresponding author.
